# Toxic stress, health and nutrition among Brazilian children in shelters

**DOI:** 10.1186/s12887-021-02577-4

**Published:** 2021-03-06

**Authors:** Adriana César da Silveira, Álvaro Jorge Madeiro Leite, Poliana Coelho Cabral, Ariclécio Cunha de Oliveira, Keciany Alves de Oliveira, Pedro Israel Cabral de Lira

**Affiliations:** 1 Nutrition PostGraduate Program at Federal University of Pernambuco, Recife, Pernambuco Brazil; 2 Department of Maternal and Child Health, Federal University of Ceará, Fortaleza, Ceará Brazil; 3 Department of Nutrition, Federal University of Pernambuco, Recife, Pernambuco Brazil; 4 Laboratory of Endocrine Physiology and Metabolism of the Institute of Biological Sciences of the State University of Ceará, Fortaleza, Ceará Brazil; 5 State University of Ceará, Fortaleza, Ceará Brazil

**Keywords:** Anemia, Child, Hair cortisol, Institutionalized, Stressful life events

## Abstract

**Background:**

Living in a shelter is an adverse experience that generates toxic stress. This situation can cause the dysregulation of the hypothalamic-pituitary-adrenal axis and exert a negative impact on health.The aim of the present study was to determine the association between toxic stress and social, clinical and nutritional characteristics in children at welfare institutions in a city of northeastern of Brazil.

**Methods:**

An analytical, cross-sectional study was conducted with male and female children up to 60 months of age who live in shelters. Hair cortisol was used for the assessment of stress (immunoassay). The anthropometric data collected were height for age, body mass index for age, arm circumference for age, and head circumference for age (expressed in z-scores). We also evaluated food intake using markers proposed by the Brazilian Dietary and Nutritional Vigilance Surveillance System as well as the occurrence of dental caries and anemia.

**Results:**

Sixty-three children one to 60 months of age participated in the present study. Asthma was the most frequent disease (11.1%). The prevalence of short stature, anemia and dental caries in the sample was 22.2, 22.2 and 9.4%, respectively. Cortisol levels ranged from 0.93 pg/mg to 391.29 pg/mg (median: 6.17 pg/mg). Higher cortisol levels were found in children with illnesses (*p* = 0.012) and those who had been hospitalized after being admitted to the institutions (*p* = 0.001).

**Conclusions:**

The majority of children had unhealthy eating behavior. The cortisol concentrations found in the present study were suggestive of dysregulation of the hypothalamic-pituitary-adrenal axis. Hypercortisolism was associated with illness and hospitalization.

## Background

Although a protection measure, being sheltered in an institution involves the breaking of family ties and affective deprivation and is therefore considered a *stressful life event* or *adverse* experience [1, 2]. Living in a shelter can lead to emotional problems and some children arrive at the shelter with health problems [1, 3]. Results of the Bucharest Early Intervention Project in Romania revealed that children in shelters had more symptoms, disorders and disabilities than children who had never be exposed to institutionalization [3, 4].

Intense or prolonged stress is denominated *toxic stress*. When this occurs early in life, it can exert a negative impact on brain structure due to the toxicity of the substances released, such as cortisol, which compromises mental health and can cause irreversible harm to neurodevelopment [5–9]. These adverse experiences in childhood are associated with learning difficulties and behavioral problems, which may be related to sex, genetic factors and extrinsic factors [6, 8, 10]. A positive association has been found between stress in early life and aggressivity in childhood [11]. Studies have also shown an association between stress and obesity [12, 13].

Stress can be assessed by the concentration of cortisol in different biological matrices. Cortisol measured in hair is a biomarker of chronic or toxic stress and its concentration corresponds to the activation of the stress response system – the hypothalamic-pituitary-adrenal (HPA) axis [14], which is the main mediator of the consequences of stress to the structure of the brain [15]. A significant increase in hair cortisol has been found in children after beginning school [16], in victims of abuse [17] and in situations of food insecurity [18].

## Methods

As children in shelters are exposed to toxic stress, the aim of the present study was to evaluate the association between this stress and social, clinical, and nutritional characteristics in children living in shelters in the city of Fortaleza, Brazil.

An analytical, cross-sectional study with a quantitative approach was conducted involving children up to 60 months of age living in welfare institutions in a city of the northeastern of Brazil, between May and August 2017. All 78 children in this age group sheltered in the city (according to data from the National Registry of Sheltered Children) were selected for the study. The exclusion criteria were a lack of information on the date of birth, physical disabilities and genetic syndromes.

The data were collected with the aid of structured questionnaires through interviews with the staff at the shelters and transcripts of individual files. Data collection was performed by a healthcare team composed of a nutritionist, nurse, dentist and undergraduate student of nutrition. The following variables were collected: sex, age, reason for being sheltered, length of time at the institution, history of being sheltered, dental caries, illnesses, hospitalizations after admission to the shelter, the use of corticoids, anemia, hair cortisol, weight, height, arm circumference (for individuals older than 2 years of age), head circumference (for individuals younger than 2 years of age), markers of food intake, siblings at the same shelter, contact with the mother, maternal mental disease and drug use by the mother.

The reasons for being sheltered at the institution were classified according to the categorization of the Management Department of the Social Assistance System of the Ministry of Social Development: intra-family violence (physical or psychological), sexual abuse, sexual exploitation, negligence or abandonment [19]. For the present study, all forms of violence (physical, psychological and sexual) were grouped into a single category. The main reason that led to being placed into a shelter was used for the purposes of analysis.

The dental examination was performed by a dentist and involved the cleaning of the teeth, the use of a tongue depressor and a light source, with the assistance of another researcher. The children were examined in the supine position. Dental caries experience was recorded using the dmft index, which indicates decayed teeth (d), missing teeth with an indication of extraction (m) and filled teeth (f).

The anthropometric data were those recommended by the Brazilian Dietary and Nutritional Vigilance System [20]. All children were weighed prior to the second meal of the day (just before 9 AM). Due to the routine of the institutions, most children received meals every 3 hours, which impeded weighing after fasting. The anthropometric measures were determined twice by the same evaluator and the mean was recorded. When the two weight readings differed by more than 100 g and height differed by more than 1 cm, the measurements were repeated.

Children younger than 2 years of age were weighed unclothed on a digital pediatric scale (Mobile Baby ELP-25BB, Balmak) with a capacity of 25 kg and precision of 1 g. Children older than 2 years of age were weighed in undergarments on a digital scale (New BK-F/FA, Balmak) with a capacity of 150 kg and precision of 100 g (platform: 40 × 40 cm; non-slip surface).

The stature of children younger than 2 years of age was determined with the child lying on a flat surface and was measured with the aid of 100-cm wooden pediatric ruler with a precision of 1 cm. The stature of children older than 2 years of age was measured with the child in the standing position with the aid of a retractable aluminum ruled installed in the column of the scale with a capacity of 2 m and precision of 0.5 cm. Arm and head circumference were measured using a plastic, non-flexible metric tape graduated in millimeters.

The indices used for the anthropometric evaluation were stature for age (S/A), body mass index for age (BMI/A), head circumference for age (HC/A) and arm circumference for age (AC/A) according to sex and expressed in z-scores. The Anthro v3. 2.2 program [21] was used for the classification of nutritional status using the cutoff points recommended by the World Health Organization (WHO) and adopted by the Dietary and Nutritional Vigilance System: for the BMI/A index, a z-score < − 2 indicates undernourished/underweight, z-score ≥ − 2 and ≤ + 1 is the ideal range, z-score > + 1 and ≤ + 2 indicates a risk of overweight and z-score > + 2 indicates excess weight (overweight and obesity); for the S/A index, a z-score − 2 indicates short stature and z-score ≥ − 2 indicates adequate stature. WHO values were used for the classification of birthweight: < 2500 g = low weight; 2500 to 2999 g = insufficient weight; 3000 to 3999 g = adequate weight; and > 4000 g = macrosomia [22].

The “Food Intake Markers” proposed by the Dietary and Nutritional Vigilance System was used for the evaluation of food intake. The classification and analysis of the food intake data were based on the “Guidelines for the Assessment of Food Intake Markers in Primary Care” of the Brazilian Health Ministry, which recommends the division by age group: up to 5 months and 29 days; 6 to 23 months and 29 days; and ≥ 2 years [23]. The markers enable defining healthy and unhealthy eating behavior. The consumption of fruit, vegetables and beans is considered a marker of healthy eating, whereas the consumption of ultra-processed foods (processed meats, sweetened beverages, instant pasta, crackers, sweets and cookies with filling) is considered a marker of unhealthy eating. At the institutions, the employees responsible for the direct care of the children worked in shifts; thus, the food consumption form was used to evaluate the frequency of consumption.

Anemia was determined by the concentration of capillary hemoglobin (Hgb) using the *HemoCue* portable photometer for direct reading in 10 μl of blood obtained using disposable lancets. In children younger than 1 year of age, the drop of blood was collected from the side of the heel with the puncture performed vertically after disinfection with 70% alcohol and drying of the skin surface with cotton. In children older than 1 year of age, the puncture was performed on the side of the tip of one of the fingers after washing with soap and water, disinfection with 70% alcohol and complete drying. Hgb < 9.5 g/dL in children less than 6 months of age and Hgb < 11 g/dL in children six or more months of age were considered indicative of anemia [24].

Stress was evaluated by the determination of hair cortisol following the Catherine Herba protocol and the criteria of the Society of Hair Testing [25]. Strands of hair measuring at least 3 cm were collected from the posterior vertex of the scalp, with 20 mg of strands used from each child. Cortisol extraction and analysis were performed at the Endocrine and Metabolism Physiology Lab of the Institute of Biological Sciences of the State University of Ceará following the method adopted by other authors [26, 27]. The analysis was performed using an enzyme-linked immunosorbent assay (ELISA) kit (Quality Control Sheet – Cayman Chemical) following the manufacturer’s instructions. Cortisol concentrations were expressed as pg/mg.

The statistical analysis was performed with the aid of the SPSS program, version 22.0 (SPSS Inc., Chicago, IL, USA). The Kolmogorov-Smirnov test was used to determine the distribution (normal or non-normal) of the continuous variables. Data with normal distribution were expressed as mean and standard deviation values and those with non-normal distribution were expressed as median and interquartile range. The comparison of means was performed using the Student’s t-test (two means) and ANOVA (two or more means). Either the chi-square test (χ^2^) or Fisher’s exact test was used for the comparison of frequencies. Analysis of covariance (ANCOVA) was used for the analysis of factors associated with mean concentrations of hair cortisol (dependent variable). The independent variables (associated factors) selected for the multivariate analysis were those with a *p*-value < 0.20 in the bivariate analysis. Variables with a *p*-value < 0.05 in the final model were considered significantly associated with the dependent variable.

This study was conducted in accordance with the ethical principles stipulated in the Declaration of Helsinki and the norms for research involving human subjects stipulated in Resolution 466/12 of the Brazilian National Board of Health. The study received approval from the Human Research Ethics Committee of the Federal University of Pernambuco (protocol number: 2.019.560, 18 April 2017; certificate number: 64680116.4.00005208). The statement of informed consent and letter of authorization were signed by the coordinating judge of the Children’s District Court of Fortaleza.

## Results

One shelter declined to participate in the study and three children were excluded (one with microencephaly and two with cerebral palsy). Thus, the data on 63 children from one to 60 months of age were analyzed. However, only 49 children were included in the study of hair cortisol, as the other 11 children had an insufficient quantity of hair or strands less than 3 cm. Girls accounted for 52.4% of the samples and age ranged from one to 60 months (mean: 30.0 ± 16.4 months). The main reasons for being admitted to a shelter were negligence (47.6%) and abandonment (36.5%). Ten children (15.9%) had been living at the institutions for more than 2 years. Age of the children at the time of being admitted to the shelter ranged from 0 to 56 months; four (6.6%) were newborns and 35 (55.5%) were children between one and 24 months of age. A total of 36% of the children had previously been at another shelter. Only 27% of the children had contact with their mothers and 44.4% had siblings at the same institution. Seven (11.1%) of the mothers had a mental disease. A total of 50.8% of mothers were users of psychoactive drugs. However, this figure could be higher, as 20 charts (31.7%) had no information regarding drug use by the mother.

Regarding health status, 27% of the children had some disease. The use of oral or inhaled corticoids (alone or combined) was found in 46% of the sample. The prevalence of dental caries was 9.43%, with a dmft index of 0.28 in the population. Dental caries was not associated with any of the other variables studied. Three children refused to undergo the dental examination. Seven had no teeth and the age of these children ranged from one to 11 months. Anemia was associated with the duration of time in the shelter; the frequency was higher among children with up to 12 months at the shelter (*p* = 0.024). The data on the health status of the children are displayed in Table 1.

**Table 1:** Health conditions of children residing at welfare shelters in the city of Fortaleza, Brazil, 2017

Health conditions	N	%
**Hospitalization after admission to shelter (*n*** **= 63)**		
Yes	15	23. 8
No	48	76.2
**Health status (*n*** **= 63)**		
Healthy	46	73.0
Ill	17	27.0
Asthma	7	11.1
Cow’s milk protein allergy	2	3.2
Lactose intolerance	2	3.2
Chronic intestinal constipation	1	1.6
Wheezing infant syndrome	1	1.6
OS ASD^a^ with left-to-right flow	1	1.6
Congenital syphilis	1	1.6
Hydrocele	1	1.6
Gastroesophageal reflux disease	1	1.6
**Dental caries** ^**b**^ **(*n*** **= 53)**		
Yes	5	9.43
No	48	90.57
**Anemia (*n*** **= 62)**		
Yes	14	22.6
No	48	77.4

^a^*Ostium secundum* atrial septum defect (acyanotic congenital heart defect) without hemodynamic repercussions in this child; ^b^nly children with dentition included in calculation;

Source: Author

The frequency of short stature was 22.2%. Thirteen children (20.6%) were at risk of excess weight and eight (12.7%) had excess weight according to the BMI/A index. AC/A was considered normal in all 33 children evaluated. HC/A was below the − 2 z-score in seven (26.9%) among the 26 children evaluated. Regarding food intake, 38 (61.3%) of the children had unhealthy eating behavior.

Cortisol concentration exhibited non-normal distribution and ranged from 0.93 to 391.29 pg/mg (median: 6.17 pg/mg; interquartile range: 7.26). For the statistical analyses, the highest value was excluded because it was very distant from the median. Thus, in the analysis performed with 48 children, cortisol ranged from 0.93 to 54.78 pg/mg. The quartile values were Q1 = 3.2, Q2 = 6.0 and Q3 = 10.4. In the ANCOVA controlled for the use of corticoids, no statistically significant differences were found among age groups (Table 2).

**Table 2:** Concentration of hair cortisol per age group of children residing at welfare shelters, Brazil, 2017

Age group	N	Cortisol (pg/mg)		***p***-value	Adjusted ***p***-value ^**†**^
		Mean	Standard deviation		
0 12 months	8	15.82	± 13.86	0.080	0.100
12 24 months	11	9.48	± 5.83		
24 36 months	13	11.74	± 15.36		
36 48 months	10	3.49	± 2.05		
48 60 months	6	3.55	± 1.51		
Total	48	9.16	± 10.83		

† *p*-value adjusted for use of corticoids using ANCOVA. Cortisol as dependent variable

Source: Author

However, positive associations were found between hypercortisolism and the occurrence of illness and hospitalization (Table 3).

**Table 3:** Analysis of covariance of hair cortisol and characteristics of children residing at welfare shelters, Brazil, 2017

VARIABLES	Categories	N	Cortisol (pg/mg)		***p***-value	Adjusted ***p***-value ^**†**^
			Mean	Standard deviation		
**Reason for admission to shelter**	Abandonment	18	10.10	10.77	0.707	
	Negligence	21	9.51	12.62		
	Violence	9	6.46	5.87		
**Stay at shelter**	≤ 12 months	33	10.38	12.52	0.251	
	> 12 months	15	6.47	4.96		
**Past history of shelter**	Yes	35	9.24	9.58	0.936	
	No	13	8.95	14.12		
**Age upon admission**	≤12 months	20	10.57	9.81	0.361	
	> 12 months	24	7.48	12.02		
**Siblings in shelter**	Yes	24	8.64	11.45	0.742	
	No	24	9.68	10.40		
**Contact with mother**	Yes	12	8.85	10.17	0.910	
	No	36	9.26	11.18		
**Illness**	Yes	11	15.83	18.40	0.155	0.012*
	No	37	7.18	6.45		
**Hospitalization**	Yes	11	19.16	18.09	0.039*	0.001*
	No	37	6.18	4.76		
**Caries**	Yes	2	4.72	1.82	0.559	
	No	46	9.35	11.03		
**Anemia**	Yes	10	9.92	13.34	0.831	
	No	37	9.08	10.39		
**Eating behavior**	Healthy	19	8.63	12.20	0.788	
	Unhealthy	29	9.51	10.05		
**Stature for age**	Low	10	10.36	13.45	0.698	
	Adequate	38	8.84	10.23		

† *p*-value adjusted for use of corticoids using ANCOVA. Cortisol as dependent variable

*Only significant variables (*p* ≤ 0.20) included in analysis

Source: Author

## Discussion

The present results enable us to infer that stress had a negative impact on the health of the sample studied. The most prevalent disease was asthma, which is the main cause of hospitalization among Brazilian children [28] and is positively associated with stress [9, 29]. Contrasting the findings of this study, KAMPS et al. (2014) [30] conducted a case-control study evaluating the effect of asthma and its treatment on the HPA axis and found that the hair cortisol levels of the children with asthma were significantly lower than those of the healthy controls. However, children living in shelters are exposed to numerous stressors related to their past situations as well as those inherent to institutionalization.

Toxic shock may be the trigger for the manifestation of genetically programmed diseases [31]. As hospitalization is an adverse experience [32], becoming ill can initiate a cycle of stressful experiences in children living in a situation of welfare institutionalization. Few children had protective factors, such as contact with their mothers and sibling at the same institution. Not all institutions have psychologists on staff and none had the support of religious groups on a permanent basis.

The illnesses found in the population studied are highly prevalent in this age group. Therefore, disease prevention and control and a reduction in the stay at a shelter could avoid or reduce stress and its negative consequences, which impede full, healthy development. The placement of these children in a foster family (modality that offers care in a family environment and ensures individualized attention) is an alternative to institutionalization, but, unfortunately, is a rare occurrence in Brazil, where the shelter remains the main modality. Foster care is ensured in Article 34 of the Child and Adolescent Statute and, according to this law, should be a priority measure for the protection of these children [33].

The Bucharest Early Intervention Project in Romania investigated the impact of psychosocial deprivation associated with institutional child care in early life and the findings suggest that early psychosocial negligence can result in cognitive deficits, whereas children under the care of a foster family develop better [34]. Therefore, the institutionalization of children can lead to diseases and neurodevelopmental disorders that can exert a long-term impact on the lives of these children and even influence future generations.

The cortisol data did not exhibit normal distribution, which is similar to findings described in previous studies [14, 35, 36]. Some children had a concentration lower than that found in the literature and, in the analysis of quartiles, some had concentrations lower than the 1st quartile (3.2), suggesting hypocortisolism. As toxic stress leads to the dysregulation of the HPA axis, it can cause either hypercortisolism or hypocortisolism [37, 38]. Although the mechanism by which hypocortisolism occurs in toxic stress has not yet been clarified, hypotheses suggest that the hyperresponsivity of the HPA makes it hyporesponsive or there may be hypersensitivity of the target tissue [9, 10, 39]. Moreover, exposure to excessive maternal stress in the prenatal period has been associated with a reduction in cortisol in the hair of newborns [40].

There is evidence of hypocortisolism related to toxic stress in children deprived of their mother’s care, individuals having suffered trauma [10] and individuals with anxiety disorders [41]. Differences in the activation response of the HPA axis and, consequently, hair cortisol levels may be explained by individual differences in perceptions and psychological responses to stress due to diverse factors, such as a genetic predisposition and past traumas [6, 31].

The results regarding nutritional status are similar to those described in previous studies involving institutionalized children independently of the indicator used for the evaluation [42–44]. Among the nutritional disorders found, short stature is a common occurrence and was three times more frequent in the present investigation compared to the findings of a large population-based survey conducted in Brazil with non-institutionalized children [45]. This reveals that factors related to living in a shelter may exert an effect on aspects that are indispensable to normal growth, such as nutrition and health care.

Although the prevalence has been declining, short stature remains one of the major nutritional disorders in Brazil [46]. The illnesses found in the present study do not have an aggressive impact on nutritional status. However, we found unhealthy eating behavior in the majority of the sample, which reveals the low quality of the menu offered.

Approximately half of the children made use of corticoids. The prolonged use of these medications can have side effects, such as linear growth deficit [47]. The use of corticosteroids is associated with a reduction in the functioning of executive cognition as well as a greater likelihood of mood disorders and anxiety in the general adult population [48]. Therefore, the prescription of such medications to populations exposed to toxic stress should be evaluated carefully.

A positive association was found between anemia and a shorter length of stay in the shelter. This finding reveals that anemia may be minimized or corrected at such institutions, as hemoglobin can take months to normalize. Although food intake in the majority of children at the institutions was not considered adequate, it may have been better than that prior to institutionalization, enabling an improvement in hemoglobin levels.

This is the first study on toxic stress in Brazilian children who live in shelters to evaluate hair cortisol as a marker of stress. The age group of the present sample is the same as the majority of children who live in shelters in the city of Fortaleza. Although some data were secondary, this information was collected from official documents that are valid for the entire country, such as the declaration of a live birth.

A control group of non-institutionalized and a larger sample size may have brought other significant associations to light. As chronic stress can exert a considerable biopsychosocial impact, adequately addressing the physical and mental health of institutionalized children is of the utmost importance.

## Conclusions

Some of the children in the present study had cortisol concentrations suggestive of the dysregulation of the HPA axis. The occurrence of illness and a history of hospitalization were associated with higher concentrations of hair cortisol. High frequencies of short stature and anemia were found in the population studied and the analysis of dietary habits revealed that the majority of children had unhealthy eating behavior. The present results demonstrate an urgent need to improve the nutrition and health (physical and mental) of the children studied.

## Data Availability

The datasets used and analyzed in this study are available from the corresponding author upon request.

## Abbreviations

HPA: Hypothalamic-pituitary-adrenal
BMI/A: Mass index for age
HC/A: Head circumference for age
AC/A: Arm circumference for age
S/A: Stature for age
WHO: World Health Organization
Hgb: Hemoglobin
ELISA: Enzyme-linked immunosorbent assay
ANCOVA: Analysis of covariance
dmf-t: decayed teeth (d), missing teeth with an indication of extraction (m) and filled teeth (f)
